# Algorithmic Spaced Retrieval Enhances Long-Term Memory in Alzheimer Disease: Case-Control Pilot Study

**DOI:** 10.2196/51943

**Published:** 2024-07-19

**Authors:** Amy M Smith, Anna Marin, Renee E DeCaro, Richard Feinn, Audrey Wack, Gregory I Hughes, Nathaniel Rivard, Akshay Umashankar, Katherine W Turk, Andrew E Budson

**Affiliations:** 1 Blank Slate Technologies, LLC Arlington, VA United States; 2 Center for Translational Cognitive Neuroscience, VA Boston Healthcare System Boston, MA United States; 3 Alzheimer’s Disease Research Center, Boston University Boston, MA United States; 4 Frank H Netter MD School of Medicine Quinnipiac University North Haven, CT United States; 5 The US Army Combat Capabilities Development Command (DEVCOM) Soldier Center Natick, MA United States; 6 The Center for Applied Brain and Cognitive Sciences, Tufts University Medford, MA United States; 7 Yale School of Medicine New Haven, CT United States; 8 Univerity of Texas at Austin College of Education Austin, TX United States

**Keywords:** Alzheimer disease, spaced retrieval, mobile app, assistive technology, episodic memory, semantic memory, mobile phone

## Abstract

**Background:**

Spaced retrieval is a learning technique that involves engaging in repeated memory testing after increasingly lengthy intervals of time. Spaced retrieval has been shown to improve long-term memory in Alzheimer disease (AD), but it has historically been difficult to implement in the everyday lives of individuals with AD.

**Objective:**

This research aims to determine, in people with mild cognitive impairment (MCI) due to AD, the efficacy and feasibility of a mobile app that combines spaced retrieval with a machine learning algorithm to enhance memory retention. Specifically, the app prompts users to answer questions during brief daily sessions, and a machine learning algorithm tracks each user’s rate of forgetting to determine the optimal spacing schedule to prevent anticipated forgetting.

**Methods:**

In this pilot study, 61 participants (young adults: n=21, 34%; healthy older adults: n=20, 33%; people with MCI due to AD: n=20, 33%) used the app for 4 weeks to learn new facts and relearn forgotten name-face associations. Participation during the 4-week period was characterized by using the app once per day to answer 15 questions about the facts and names. After the 4-week learning phase, participants completed 2 recognition memory tests approximately 1 week apart, which tested memory for information they had studied using the app as well as information they had not studied.

**Results:**

After using the mobile app for 1 month, every person with MCI due to AD demonstrated improvements in memory for new facts that they had studied via the app compared to baseline (*P*<.001). All but one person with MCI due to AD (19/20, 95%) showed improvements of more than 10 percentage points, comparable to the improvements shown by young adults and healthy older adults. Memory for name-face associations was similarly improved for all participant groups after using the app but to a lesser degree. Furthermore, for both new facts and name-face associations, we found no memory decay for any participant group after they took a break of approximately 1 week from using the app at the end of the study. Regarding usability, of the 20 people with MCI due to AD, 16 (80%) self-adhered to the app’s automated practice schedule, and half of them (n=10, 50%) expressed an interest in continuing to use it.

**Conclusions:**

These results demonstrate early evidence that spaced retrieval mobile apps are both feasible for people with early-stage AD to use in their everyday lives and effective for supporting memory retention of recently learned facts and name-face associations.

## Introduction

### Background

In the treatment of Alzheimer disease (AD), drugs such as cholinesterase inhibitors (ChEIs) are widely prescribed because they offer hope of symptom relief and place a low adherence burden on people living with AD and their caregivers. However, these drugs overwhelmingly demonstrate small improvements for memory outcomes [1-3] and can cause adverse side effects [4]. By contrast, a behavioral intervention called spaced retrieval results in large effect size improvements in memory for studied information for people with AD [5] but has been underused, perhaps because it can be complicated to administer in real-world contexts and populations. Herein, we present a pilot study on a novel mobile app that streamlines the delivery of spaced retrieval into the everyday lives of people with AD.

Spaced retrieval involves repeatedly recalling information (ie, retrieving) that one desires to learn after increasingly lengthy intervals of time (ie, spacing). This method reliably supports the learning and retention of the studied information across people of a variety of ages and abilities [6-8]. Obtaining the full benefits of this method involves mathematically estimating the forgetting curve for each to-be-remembered item to determine the ideal review schedule for each item [9,10]. Without this optimization, learners can quickly become disheartened by the time-consuming, brute-force approach of reviewing all items during each study session; and, specifically in a population consisting of people with AD, additional barriers include individuals’ ability to remember to adhere to a learning program [11] and their diminished ability to sustain attention for prolonged periods of time [12-14].

Today, these barriers are surmountable. Modern machine learning algorithms can provide data-driven estimations of forgetting curves, thus maximizing efficiency and reducing the time burden of spaced retrieval programs. In addition, the ubiquity of smartphones facilitates the seamless delivery of spaced retrieval to mobile devices, complete with automated engagement reminders. A mobile app made by the company Blank Slate Technologies [15] has capitalized on the efficacy of spaced retrieval, the recent developments in machine learning, and the prevalence of smartphones to deliver automated and individualized long-term memory support. The app incorporates spaced retrieval into a flashcard-style interface that aims to help people retain desired memories over periods of months and years. What is most novel about this approach is its additional use of a machine learning algorithm that tracks individual forgetting curves to provide individualized schedules of spacing for each user. More detail regarding the mobile app can be found in Multimedia Appendix 1.

### Objectives

The purpose of this study was to determine the efficacy and usability of this mobile app for supporting long-term retention of episodic and semantic memories in people with mild cognitive impairment (MCI) due to AD, who historically have needed researcher or caregiver support with spaced retrieval regimens [5]. Briefly, episodic memory pertains to recalling events and details that are linked to the specific context in which they occurred, whereas semantic memory refers to general knowledge of facts, language, and concepts that are not associated with a specific context. These 2 types of memory rely on different although not mutually exclusive neural pathways [16-18]. Early-stage AD impairs both episodic [19,20] and semantic [21-23] memory.

This pilot study included young adults, healthy older adults, and older adults with MCI due to AD; the young adults and healthy older adults provided a between-participants comparison of how the app influences memory in people without any age-related memory changes (young adults) and in age-matched controls without AD (healthy older adults) compared to those with AD. On the first day of the study, all participants first learned new episodic information (facts about the country Georgia) and identified previously learned semantic information (celebrity name-face associations). During the next 4 weeks, participants used the app to study half of the information from each stimulus set, providing a within-participants comparison of memory for stimuli that were studied (experimental condition) versus those that were nonstudied (control condition). Of note, the *nonstudied* condition represents the status quo in adulthood: learning new information but not engaging in any intentional studying or consistent review of this information. One day and approximately 1 week after ceasing the app, participants completed a memory test consisting of questions about the stimuli they had studied using the app versus those they had not.

On both final memory tests, we hypothesized that all participants would correctly recognize more answers to questions that had been studied using the app than those that had not. We expected to observe this for both new episodic memories (facts about the country Georgia) and preexisting semantic memories (name-face associations), demonstrating the app’s utility for helping people with MCI due to AD learn new information and maintain older, fading memories. We also predicted that young adults would demonstrate better memory for all items than healthy older adults and that healthy older adults would perform better than people with MCI due to AD. In addition, we explored the following research questions (RQs) without a priori predictions:

- RQ1: If app use improves memory, are there differences in the magnitude of this benefit between the groups?
- RQ2: Does memory decay during the week that passes between the 2 final memory tests? Does this decay differ between the 3 groups?
- RQ3: To what extent does the rate of learning differ for healthy older adults and people with MCI due to AD during the 4 weeks of app use?

## Methods

### Design

**Figure 1:**
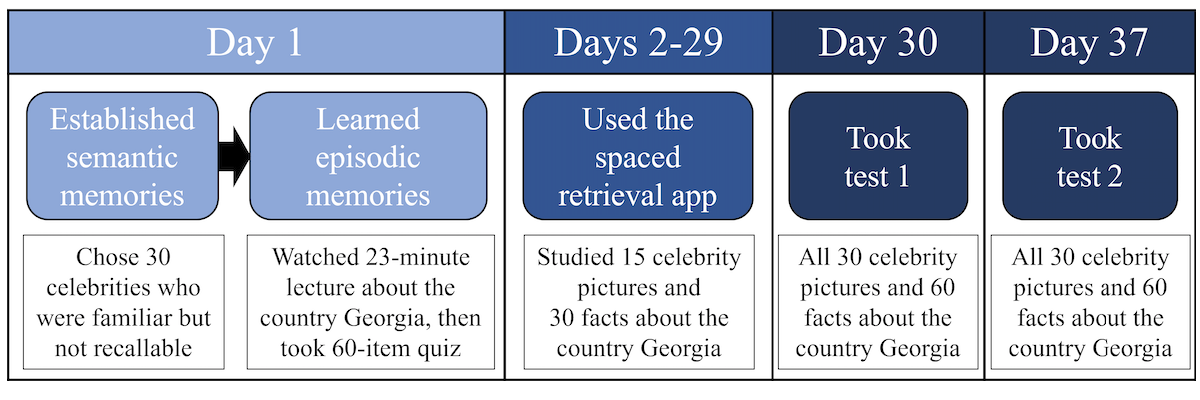
Graphic representation of the general procedure for the 5-week experiment. All participants, regardless of group, completed the same experimental procedure.

This was a case-control study featuring a 2 (*item type*: studied and nonstudied)×2 (*final test*: test 1 and test 2)×3 (*group*: young adults, healthy older adults, and people with MCI due to AD) mixed factorial design. *Item type* was manipulated within participants. Specifically, a randomly selected half of the items from the final memory test were studied during the 4-week intervention and the other half of the items were not, allowing us to compare participants’ subsequent performance on studied versus nonstudied items on the final memory tests. *Final test* was also manipulated within participants. Specifically, each participant completed both test 1 and test 2 at the end of the experiment, allowing us to compare their performances on these 2 tests that were administered approximately 1 week apart. Figure 1 presents a depiction of the procedure, which details how we manipulated studied versus nonstudied items (*item type*) and the 2 temporally segregated final memory tests (*final test*).

### Ethical Considerations

This study was approved by the Quinnipiac University Institutional Review Board (05321), the Boston University Medical Center Institutional Review Board (H-26786), and the Department of Veterans Affairs (VA) Institutional Review Board (1577783). Informed consent was obtained from all participants. Researchers obtained consent from young adults via email by sending them an electronic consent form and asking them to reply by confirming their intent to participate in the study. Researchers consented healthy older adults and people with MCI due to AD over the telephone. All data presented in this paper and Multimedia Appendices 1 and 2 have been deidentified. Participants were compensated US $100 via a payment app or check for their participation in the 5-week experiment.

### Power Analysis

We conducted a power analysis to determine the sample size needed to power our main comparison of interest: in people with MCI due to AD, the within-participants difference in recognition memory for information that was studied using the mobile app versus information that was not studied. In prior research on this app that used nearly identical methods and materials as this study, researchers found that participants who used the app for 4 weeks demonstrated memory improvement of a very large magnitude (Cohen *d*=6.04) compared to those who did not use the app during this period [24]. Thus, for people with MCI due to AD, we anticipated a large effect size improvement when comparing memory performance for information that had been studied using the app versus information that had not been studied. We conservatively estimated an effect size of Cohen *d*=0.80, the lowest value that is still considered large [25]. With this parameter in mind and the goal of achieving 95% power, a power analysis conducted using G*Power (Heinrich Heine University) [26] recommended a sample size of 19 people with MCI due to AD.

### Participants

A total of 61 adults participated in this experiment: 21 (34%) young adults (age range: 18-22 y; n=16, 76% female), 20 (33%) older adults who were deemed neurologically healthy (age: mean 75.40, SD 7.14 y; education: mean 16.20, SD 3.07 y; n=15, 75% female), and 20 older adults who were diagnosed with MCI due to AD (age: 77.65, SD 5.67 y; education: mean 14.95, SD 3.09 y; n=2, 10% female). There was not a statistical difference in age (t_38_=1.10; *P*=.28) or years of education (t_38_=1.28; *P*=.21) between healthy older adults and people with MCI due to AD.

To be considered for the experiment, all participants had to have a smartphone or a computer with reliable internet access, a telephone number on which they could be reached, access to an email account that they checked regularly, and familiarity with using a mobile app (eg, how to open and exit an app and how to type and submit responses within an app).

Of the 20 participants with MCI due to AD, 14 (70%) were recruited through the VA Boston Memory Disorders Clinic by KWT and AEB, 5 (25%) were recruited from a patient registry actively maintained by the Boston University Alzheimer’s Disease Research Center (BU ADRC; these participants are assessed annually for their cognitive and medical status and have agreed to be contacted about new research projects), and 1 (5%) was recruited through word of mouth by a member of the research staff. All participants had a documented referral from a practicing neuropsychologist or neurologist who had evaluated and diagnosed each person with MCI due to AD using the National Institute on Aging‐Alzheimer’s Association criteria [27]. All participants reported that they continually underwent annual evaluations to verify their clinical status.

Healthy older adults were recruited through the participant pool actively maintained by the BU ADRC and through word of mouth by the research staff. The BU ADRC recruits participants through community postings in libraries, older adult centers, and websites such as Craigslist. All healthy older adults self-reported their cognitive fitness and confirmed that they underwent a cognitive evaluation at least once annually with a physician. All potential older adult participants, both healthy older adults and people with MCI due to AD, were first contacted by a member of the research staff, who then described the study and screened participants for eligibility.

At the start of the experiment, all older adults with MCI due to AD as well as those without completed a version of the Montreal Cognitive Assessment (MoCA) [28] that is compatible with remote research, called the MoCA-Blind [29]. The last 2 questions on the MoCA-Blind were not included because answers could not be verified by the administering researcher (eg, “What city are you in?”), resulting in a maximum MoCA-Blind score of 20 points. Healthy older adults outperformed people with MCI due to AD on the modified MoCA-Blind (healthy older adults: mean 17.70, SD 2.00; people with MCI due to AD: mean 13.90, SD 3.60; t_38_=4.13; *P*<.001).

Young adult participants were required to be aged between 18 and 25 years and were recruited from the student body at Quinnipiac University. Participants were recruited via campus flyers posted in dining halls and academic buildings, as well as by word of mouth from the undergraduate research assistants who aided in data collection for this study. Young adults expressed their initial interest in participating by sending an email to the research laboratory run by AMS. There were no additional inclusion criteria for young adults beyond age, access to a computer or smartphone, and familiarity with mobile apps.

### Materials

#### Episodic Memory Stimuli

To form new episodic memories, participants viewed a 23-minute teaching video on the country Georgia, which was used in a prior study on the efficacy of this app [24]. Georgia was chosen as the subject matter to minimize participants’ prior knowledge. Information contained in the video came from *The World Factbook*, a reference resource produced by the Central Intelligence Agency [30]. A set of 60 four-alternative forced-choice questions was created to test participants’ knowledge of the video. The questions assessed distinct facts about topics such as Georgia’s geography, history, culture, economy, and political system.

#### Semantic Memory Stimuli

A set of stimuli representing preexisting semantic memories was constructed for each participant. At the start of the study, participants viewed 301 photographs of celebrities (eg, American actress and producer Reese Witherspoon) and were asked to indicate whether they (1) could recall the name, (2) could not recall the name but would recognize it on a list, or (3) did not know the name. Three research assistants and authors AMS, GIH, NR, and RF constructed the set of celebrity pictures and an accompanying 4-item forced-choice recognition test. Celebrities were chosen based on their popularity during the period spanning 1950 to 2020. The chosen photographs represented each celebrity at the time of their peak popularity. The foils on the multiple-choice test were also celebrities and were chosen based on sharing age, gender, general appearance, and media genre in common with each target.

### Procedure

Figure 1 presents a graphic depiction of the general procedure. Researchers contacted interested participants by telephone (older adults) or email (young adults). After providing informed consent, the older adults completed the modified MoCA-Blind, answered standard demographic questions, and were screened for having normal or corrected-to-normal vision (all participants responded affirmatively). Young adults completed informed consent and the demographic questionnaire via email.

All participants were next emailed a link to a Typeform [31] survey, which was used to facilitate the selection of semantic and episodic memory stimuli. Participants first viewed celebrity photographs, judging each photograph according to whether they (1) could recall the name, (2) could not recall the name but would recognize it on a list, or (3) did not know the name. Participants viewed the stimuli until they had identified 30 celebrities that they knew but could not recall (option 2). In cases where a participant viewed all 301 stimuli and did not identify 30 that they felt they knew but could not recall, this participant was assigned the rest of their stimuli from the bank of questions for which they indicated they did not know the answer; for example, if a participant viewed all 301 celebrity photographs and selected option 2 only 22 times, they were randomly assigned 8 additional questions for which they had chosen option 3. Of the 61 participants, 16 (26%; young adults: n=7, 44%; healthy older adults: n=5, 31%; people with MCI due to AD: n=4, 25%) did not identify 30 familiar celebrity faces from the stimulus set and were thus assigned to view photographs of some celebrities that they indicated they did not know (young adults: mean 11.71, SD 6.1; healthy older adults: mean 12.00, SD 7.8; people with MCI due to AD: mean 17.25, SD 6.9).

The Typeform survey next invited participants to take a break if needed. They were then instructed to watch the 23-minute lecture on the country Georgia, followed by another invitation to take a break. Finally, participants completed the 60-item multiple-choice test about the video.

Within 1 week of completing the Typeform survey, participants were contacted via email (young adults) or telephone (older adults) with instructions about how to access the app on their smartphone or computer. Instructions included how to download the app, log in using provided credentials, and opt in for email and push notification alerts. Participants recruited through Quinnipiac University and Boston University provided their personal email addresses for creating their accounts. Once participants had established their accounts and confirmed their comfort with using the app, the researcher instructed them to be on the lookout for push notifications and emails reminding them to answer their app questions over the next 4 weeks. Participants recruited through the VA used a randomly coded dummy email address for their accounts, in accordance with local institutional review board regulations. These participants received encrypted email reminders from research-credentialed VA staff.

During the following 4 weeks, participants were prompted via push notification and email at spaced intervals to open the app and engage in self-testing for half (15/30, 50%) of the celebrity faces and half (30/60, 50%) of the Georgia questions for a total of 45 questions. The 15 celebrity photographs were chosen randomly from each participant’s 30-item stimulus set, and the 30 Georgia questions were assigned according to 1 of 4 counterbalances from the full 60-item set. The 45 items that were not studied during this 4-week period served as a baseline comparison on the final memory test. Participants were limited to 15 questions per day to avoid fatigue effects, and they were able to choose, through the app’s settings, what time they received their notifications each day. The frequency of notifications to engage with the app ranged from every 1 to 5 days (mean 1.43, SD 0.91 d) depending on the algorithm’s estimation of each person’s knowledge levels, except for participants recruited through the VA, who received daily encrypted email reminders as described previously. During the 4-week period, the research team monitored each participant’s engagement with the app using Blank Slate Technologies’ analytics dashboard, which is accessible via a web browser. If participants did not use the app for 3 consecutive days, a researcher emailed participants or contacted them by telephone on the fourth day to remind them to use it. Young adults and healthy older adults adhered to using the app on their own, whereas some of the older adults with MCI due to AD (4/20, 20%) were assisted by caregivers throughout the study. For a more detailed description of the mobile app used in this research, please refer to Multimedia Appendix 1. The mobile app used in this experiment is available for interested parties to download in the Apple App Store (under the name Braintrust: Memory Companion.

After 4 weeks of app use, the participants’ 45 stimuli were deleted from their app accounts, and test 1 was loaded in. Within 24 to 72 hours of this change, a researcher emailed each participant or contacted them by telephone and asked them to use the app to complete test 1, a multiple-choice memory test containing all 90 questions: 30 celebrity face recognition questions and 60 questions about the country Georgia. Of note, half of each stimulus set had been reviewed during the prior 4 weeks. On average, participants completed test 1 within 3.38 (SD 2.88) days of ceasing the app. Test 1 took approximately 30 minutes to complete.

Approximately 1 week (a minimum of 7 days) after participants completed test 1, a researcher again emailed participants or contacted them by telephone and asked them to use the app to complete test 2, the final multiple-choice memory test including all 90 questions. On average, participants completed test 2 within 8.30 (SD 1.99) days of finishing test 1. After finishing test 2, a researcher contacted participants to thank them, debrief them, and coordinate payment.

### Data Scoring

#### Episodic Memory

For the baseline memory test (60 questions) and the 2 final memory tests (60 questions each), all responses to questions about the country Georgia were coded as 0 (incorrect) or 1 (correct). We calculated the proportion of accurate responses for each participant for each test. For the 2 final memory tests, separate proportions were computed for studied and nonstudied items. Thus, proportions were calculated out of 60 items for the baseline test and out of 30 items for the studied and unstudied portions of each of the 2 final memory tests.

#### Semantic Memory

Of note, there was not a baseline measure of semantic memory. For the 2 final memory tests (30 questions each), all responses to celebrity-recognition questions were coded as 0 (incorrect) or 1 (correct). We calculated the proportion of accurate responses for each participant for each test. Separate proportions were computed for studied and unstudied items. Thus, proportions were calculated out of 15 items for the studied and unstudied portions of each of the 2 final memory tests.

#### Learning Curves

Traditional learning curves could not be calculated because the app’s spaced retrieval algorithm ensures that participants do not review all items during each learning session. Instead, we estimated content mastery over time by calculating cumulative accuracy proportions for each participant on each of the 28 days, that is, for each day of the study, we calculated each participant’s accuracy on all their most recent responses to each of their 45 questions; for example, a participant’s accuracy score on day 17 of the study would be calculated by searching for their most recent viewing of each of the 45 questions and then calculating the proportion of those 45 questions that were answered accurately.

#### App Use Statistics

To examine group differences in app use statistics during the learning phase, we computed for each participant the (1) total number of app sessions, (2) total number of questions reviewed on the app, (3) total number of minutes spent using the app, (4) average number of seconds spent reviewing each question on the app, (5) average number of viewings per question, and (6) average number of days that passed between app sessions (ie, each participant’s spacing interval). Regarding the second of these variables—total number of questions reviewed on the app—it should be noted that all participants were assigned 15 questions to review in each session, but they had to attempt each question as many times as necessary until they answered it correctly. Thus, the total number of questions reviewed could vary depending on each participant’s accuracy.

### Analysis Plan

#### Episodic Memory

We conducted a 1-way ANOVA to explore differences between the groups for baseline recognition memory for episodic information (facts about the country Georgia). We also conducted a 2 (*item type*: studied and nonstudied)×2 (*final test*: test 1 and test 2)×3 (*group*: young adults, healthy older adults, and people with MCI due to AD) factorial ANOVA on recognition memory for episodic information. Of the 20 participants with MCI due to AD, 1 (5%) was excluded from this analysis because they did not complete the baseline test.

#### Semantic Memory

We conducted a 2 (*item type*: studied and nonstudied)×2 (final test: test 1 and test 2)×3 (*group*: young adults, healthy older adults, and people with MCI due to AD) factorial ANOVA on recognition memory for semantic information (celebrity names).

#### Learning Curves

All learning phase analyses compared healthy older adults and people with MCI due to AD. We used a nonlinear exponential growth mixed model [32] to model the change in scores during the 28 days of app use. The model contains 3 parameters: an asymptote representing maximum performance at the end of use; change, representing the amount of change in score from day 1 to the asymptote; and rate, representing how fast the scores change over time and reach the asymptote. Differences in the 3 parameters were tested between healthy older adults and people with MCI due to AD.

#### App Use Statistics

We conducted independent samples 1-tailed *t* tests to examine group differences between healthy older adults and people with MCI due to AD in terms of the (1) total number of app sessions, (2) total number of questions reviewed during the learning phase, (3) total number of minutes spent using the app, (4) average number of seconds spent reviewing each question, (5) average number of viewings per question, and (6) average number of days that passed between app sessions (ie, each participant’s spacing interval).

#### Usability and Feasibility

Throughout the experiment, the researchers involved in data collection recorded qualitative notes from conversations with older adult participants regarding their comfort with using the app. The researchers also recorded the number of older adults who needed to be contacted at least once to be reminded to use the app.

## Results

### Episodic Memory

On the initial baseline test of episodic memory (day 1 of the experiment), we found a main effect of *group* (*F*_2,57_=6.04; *P*=.004; η_p_^2^=0.18). Bonferroni post hoc comparisons showed that young adults (mean 0.63, SEM 0.03) and healthy older adults (mean 0.61, SEM 0.03) did not differ in their baseline recognition memory for the Georgia video (t_39_=0.34; *P*=.99). However, people with MCI due to AD (mean 0.49, SEM 0.03) had lower recognition performance than both young adults (t_39_=3.20; *P*=.007; Cohen *d*=1.01) and healthy older adults (t_38_=2.83; *P*=.02; Cohen *d*=0.91).

**Figure 2:**
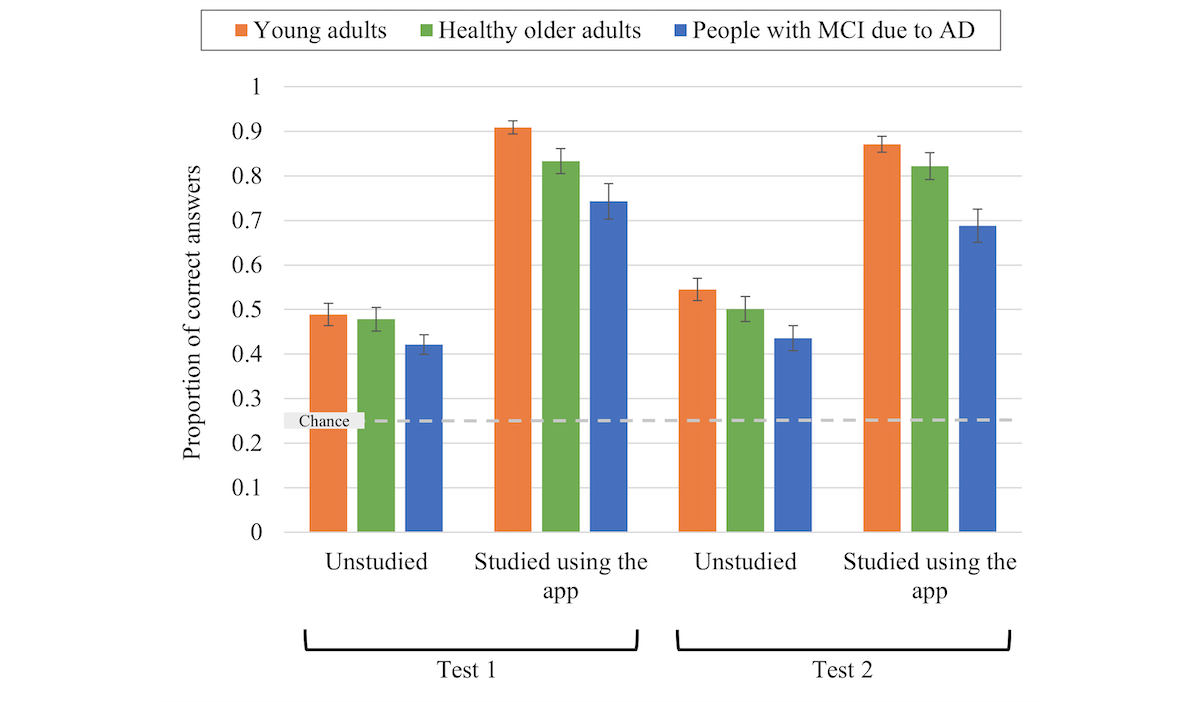
Recognition memory performance on the final episodic (facts about the country Georgia) memory tests, test 1 and test 2. Error bars represent SEMs. AD: Alzheimer disease; MCI: mild cognitive impairment.

Figure 2 presents a graphic depiction of the results from the final episodic memory tests, test 1 and test 2. Our factorial ANOVA revealed main effects of *item type* (*F*_1,58_=603.10; *P*<.001; η_p_^2^=0.91) and *group* (*F*_2,58_=8.61; *P*<.001; η_p_^2^=0.23), as well as a significant *item type*×*group* interaction (*F*_2,58_=3.40; *P*=.04; η_p_^2^=0.11). Post hoc tests using the Holm-Bonferroni correction showed that, for all 3 groups, recognition memory was better for studied items than for unstudied items (all *P* values <.001), and this held true when comparing between groups (all *P* values <.001); for example, notably, young adults’ memory for unstudied items was significantly lower than older adults’ memory for studied items, indicating that using the app helped both older adult groups outperform young adults when young adults did not use the app (healthy older adults versus young adults: t_39_=8.59; *P*<.001; Cohen *d*=2.48; people with MCI due to AD versus young adults: t_39_=5.50; *P*<.001; Cohen *d*=1.58).

The interaction was driven by *group* differences within each *item type*. On unstudied items, there were no group differences in recognition performance (no *P* value met the threshold for significance). On studied items, young adults and healthy older adults’ recognition performance did not differ (*P*=.26), but people with MCI due to AD had lower recognition performance than both young adults (t_39_=4.83; *P*<.001; Cohen *d*=1.39) and healthy older adults (t_38_=3.05; *P*=.02; Cohen *d*=0.89).

Although we did not find a main effect of *final test* (test 1 vs test 2; *P*=.85), our ANOVA did return a significant *item type*×*final test* interaction (*F*_1,58_=21.25; *P*<.001; η_p_^2^=0.27). This interaction was driven by changes in performance from test 1 to test 2 within each *item type*. Unstudied items were recognized at higher rates on test 2 than on test 1 (t_88_=2.52; *P*=.01; Cohen *d*=0.25), whereas studied items were recognized at lower rates on test 2 than on test 1 (t_88_=−2.82; *P*=.01; Cohen *d*=0.28). This finding is considered an artifact and is not discussed further. To facilitate future data exploration (eg, meta-analyses), all means and SEMs from this analysis are presented in Multimedia Appendix 2.

### Semantic Memory

**Figure 3:**
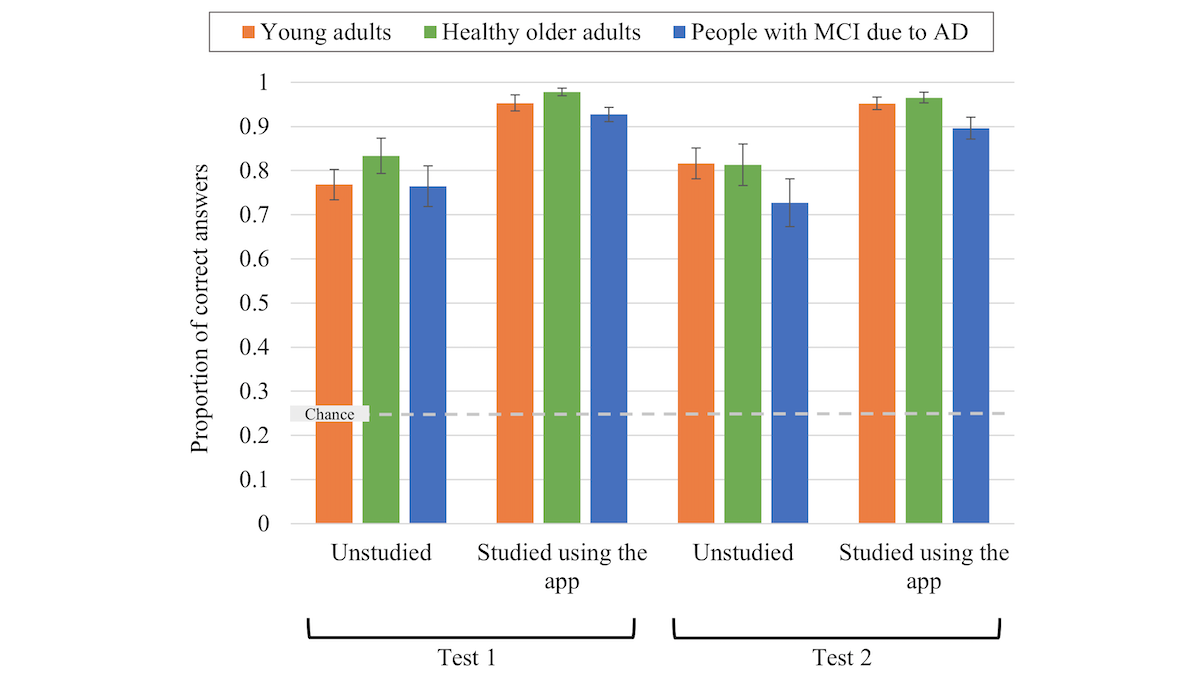
Recognition memory performance on the final semantic (celebrity names) memory tests, test 1 and test 2. Error bars represent SEMs. AD: Alzheimer disease; MCI: mild cognitive impairment.

Figure 3 presents a graphic depiction of the results from the final semantic memory tests, test 1 and test 2. Our factorial ANOVA revealed a main effect of *item type* (*F*_1,58_=49.89; *P*<.001; η_p_^2^=0.46) but no other significant effects (all *P* values >.05). All participants demonstrated better recognition memory for studied versus unstudied items, and this effect did not differ by *group* and showed no change during the 1-week interval between test 1 and test 2. To facilitate future data exploration (eg, meta-analyses), all means and SEMs from this analysis are presented in Multimedia Appendix 2.

### Learning Phase

#### Learning Curves

**Figure 4:**
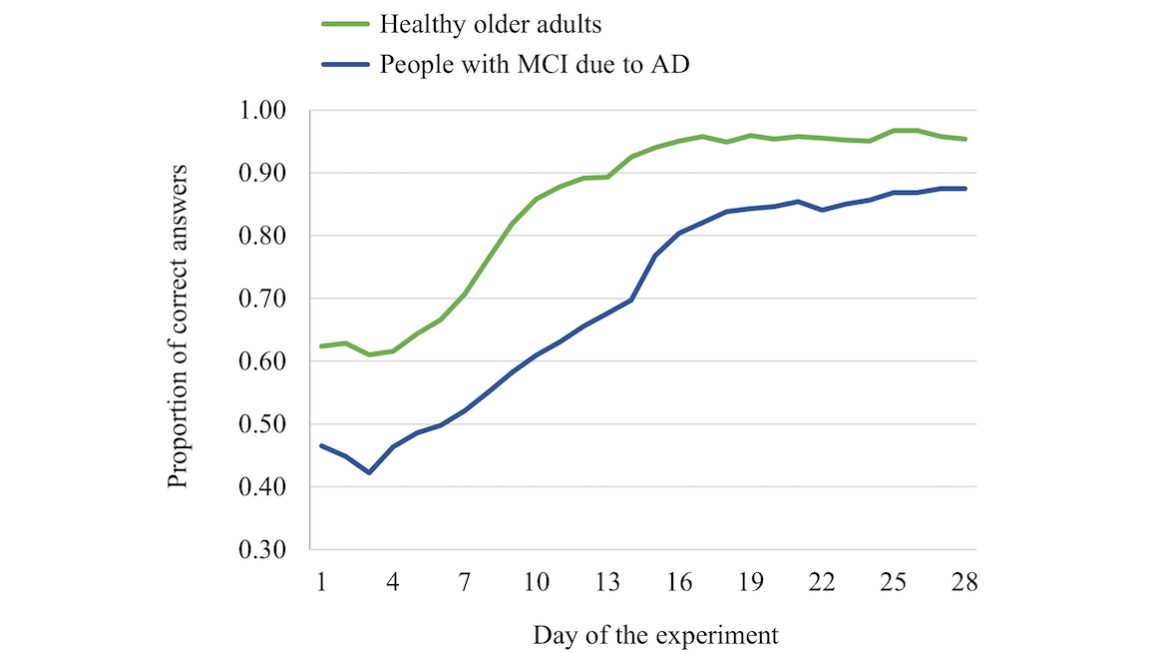
Learning curves for episodic information (facts about the country Georgia) for healthy older adults and people with mild cognitive impairment (MCI) due to Alzheimer disease (AD).

**Figure 5:**
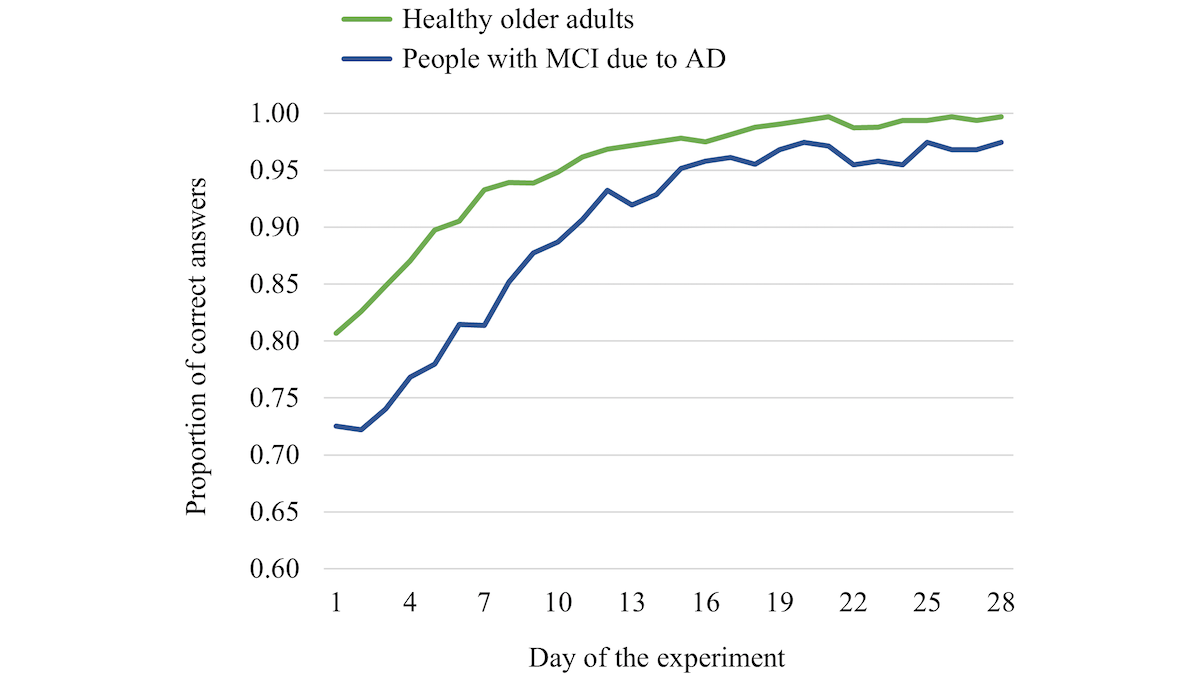
Learning curves for semantic information (celebrity names) for healthy older adults and people with mild cognitive impairment (MCI) due to Alzheimer disease (AD).

Figures 4 and 5 present graphic depictions of learning curves for healthy older adults and people with MCI due to AD. For episodic memory (Figure 4), the estimated asymptote, which is the predicted maximum score that can be achieved, was 2.68 points lower in people with MCI due to AD compared to healthy older adults but not significantly different (β=–2.68, SEM 5.01; *P*=.60). Similarly, the amount of change from day 1 to day 28 was 5.51 points higher in people with MCI due to AD compared to healthy older adults but not significantly different (β=5.51, SEM 5.99; *P*=.36). However, as evident from Figure 4, the rate of change in the curve was significantly lower in people with MCI due to AD (β=–0.12, SEM 0.01; *P*<.001). In summary, this analysis suggests that, compared to healthy older adults, people with MCI due to AD achieved statistically similar episodic knowledge levels by the end of the study, but it took them longer to accomplish comparable levels of mastery.

In comparing the semantic memory learning curves (Figure 5), the asymptote, which is the predicted maximum score that can be achieved, was 2.72 points lower in people with MCI due to AD compared to healthy older adults but not statistically different (β=–2.72, SEM 1.50; *P*=.08). The amount of change from day 1 to day 28 was estimated to be 18.31 points higher for people with MCI due to AD compared to healthy older adults, and this difference was statistically significant (β=18.31, SEM 3.96; *P*<.001). Accompanying this finding, the rate of change in the curve was significantly higher in people with MCI due to AD compared to healthy older adults (β=.15, SEM 0.02; *P*<.001). In summary, this analysis suggests that, compared to healthy older adults, people with MCI due to AD achieved statistically similar semantic knowledge levels by the end of the study, and they experienced a greater improvement in knowledge levels from the first to the last day of the study; however, they had a steeper learning curve.

#### App Use Statistics

Table 1 presents app use statistics for healthy older adults and people with MCI due to AD. In comparison to healthy older adults, people with MCI due to AD reviewed more total questions during the 4-week learning phase (t_38_=2.49; *P*=.02; Cohen *d*=0.79) and spent more total minutes using the app (t_38_=3.76; *P*<.001; Cohen *d*=1.19). People with MCI due to AD also spent more seconds reviewing each question (t_38_=3.26; *P*=.002; Cohen *d*=1.03) and averaged more viewings per question (t_38_=2.49; *P*=.02; Cohen *d*=0.79). We did not find any group differences in the total number of app sessions over the 4-week period (*P*=.31) or in the average number of days that passed between app sessions (*P*=.61).

**Table 1:** Descriptive statistics for app use metrics over the 4-week study phase during which participants used the app according to an individualized spaced retrieval schedule determined by the app.

	Healthy older adults, mean (SEM)	People with MCI^a^ due to AD^b^, mean (SEM)
Total questions reviewed	254.10 (15.70)	392.90 (53.43)
Total time (min) spent using the app	39.61 (3.52)	96.42 (14.68)
Time (seconds) spent per question	9.26 (0.56)	14.51 (1.51)
Viewings per question	5.65 (0.35)	8.75 (1.19)
Total number of app sessions	21.25 (1.24)	19.75 (0.75)
Number of days between app sessions	1.49 (0.51)	1.56 (0.42)

^a^MCI: mild cognitive impairment.

^b^AD: Alzheimer disease.

#### Change Scores

In Table 2, we combined data from both memory types (episodic and semantic) on test 1 and calculated the change score for unstudied versus studied items on the recognition test. Table 2 also displays each person’s app use data to provide a fuller picture of the effort that each person made during the learning phase and their resulting success on test 1.

**Table 2:** App use and recognition performance on test 1 for every person with mild cognitive impairment due to Alzheimer disease in our sample. The “Change score” column was added to emphasize that every participant in this group experienced a memory benefit from using the app. Participants are organized from highest to lowest change score.

	4-week study phase							Final test 1		
	Viewings per question, mean (SD)	Time per question, mean (SD)	Total time spent using the app (min)	Total number of questions answered	Total number of app sessions	Days between sessions, mean (SD)	Proportion of correct answers on nonstudied items		Proportion of correct answers on studied items	Change score

Person 1	10.47 (5.66)	28.25 (16.84)	221.80	471	23	1.23 (0.69)	0.38		0.91	+0.53
Person 2	7.51 (5.37)	7.81 (4.68)	43.98	338	25	1.17 (0.38)	0.46		0.93	+0.48
Person 3	8.07 (3.98)	25.24 (12.67)	152.72	363	23	1.23 (0.87)	0.58		0.96	+0.38
Person 4	6.24 (4.01)	19.66 (11.23)	92.08	281	18	1.00 (0.00)	0.40		0.78	+0.38
Person 5	5.36 (2.46)	8.76 (5.69)	35.17	241	20	1.47 (0.90)	0.53		0.89	+0.36
Person 6	5.44 (2.08)	13.18 (7.17)	53.82	245	20	1.47 (0.70)	0.49		0.82	+0.33
Person 7	4.56 (1.42)	7.03 (4.82)	24.02	205	18	1.47 (1.01)	0.63		0.93	+0.30
Person 8	4.64 (1.46)	7.51 (7.95)	26.17	209	14	2.23 (1.54)	0.63		0.93	+0.30
Person 9	6.04 (2.75)	22.50 (13.82)	102.00	272	17	2.19 (2.10)	0.53		0.82	+0.29
Person 10	25.93 (42.27)	11.34 (6.53)	220.48	1167	21	1.45 (0.83)	0.32		0.57	+0.25
Person 11	10.91 (9.97)	12.11 (11.32)	99.07	491	22	1.48 (0.60)	0.47		0.71	+0.24
Person 12	7.82 (3.51)	25.11 (13.84)	147.30	352	22	1.40 (0.60)	0.53		0.78	+0.24
Person 13	5.38 (2.25)	16.10 (11.45)	64.93	242	19	1.50 (0.71)	0.64		0.89	+0.24
Person 14	4.87 (2.32)	11.34 (8.42)	41.40	219	16	1.87 (1.41)	0.71		0.93	+0.22
Person 15	7.00 (7.72)	9.02 (4.78)	47.33	315	20	1.53 (0.70)	0.66		0.84	+0.19
Person 16	5.91 (2.60)	9.89 (6.38)	43.85	266	23	1.32 (0.78)	0.62		0.80	+0.18
Person 17	5.16 (3.01)	9.66 (5.49)	37.35	232	12	2.82 (1.60)	0.60		0.76	+0.16
Person 18	14.80 (12.86)	11.83 (4.99)	131.32	666	23	1.43 (0.87)	0.62		0.73	+0.11
Person 19	11.82 (9.52)	22.14 (12.58)	196.30	532	17	1.63 (0.96)	0.43		0.55	+0.11
Person 20	17.07 (16.97)	11.77 (8.38)	147.28	751	22	1.43 (0.81)	0.56		0.60	+0.04

#### Usability and Feasibility

Overall, the app seemed both easy and enjoyable to use for older adults with MCI due to AD and healthy older adults. Only 1 (5%) of the 20 healthy older adults and 1 (5%) of the 20 participants with MCI due to AD dropped out after starting using the app; the healthy adult dropped out for reasons unrelated to using the app, while the person with MCI due to AD dropped out because their caregiver found it difficult to help them adhere to app use. All healthy older adults independently adhered to the app’s intended schedule of use without needing reminder telephone calls from the research staff. Of the 20 people with MCI due to AD, 2 (10%) required 1 reminder telephone call, and 2 (10%) required multiple reminder telephone calls. Across all older participants, the only criticism of the app was that the font was too small. Half of the people with MCI due to AD (10/20, 50%) reported enjoying using the app and expressed an interest in continuing to use it.

## Discussion

### Principal Findings

In this pilot study, an algorithmic spaced retrieval mobile app delivered the memory benefits of spaced retrieval to people with early-stage AD while eliminating many previous barriers associated with the practice. After using the app for 4 weeks, people with MCI due to AD demonstrated large effect size improvements in their recognition memory for both new facts (episodic memory) and celebrity name-face associations (semantic memory), and these improvements did not decline after ceasing app use for approximately 1 week. Worthy of special emphasis, every single participant with MCI due to AD experienced memory improvement (ie, positive change scores) from using the app, suggesting that the app can benefit all people at this stage of the disease. Furthermore, of the 20 people with MCI due to AD, 16 (80%) adhered to the app’s automated schedule of spaced retrieval practice, and only 4 (20%) required reminder telephone calls. In summary, the app both supported long-term memory retention and was feasible to use for people with MCI due to AD.

We now turn to a discussion of our results in the context of our a priori hypotheses and RQs. Consistent with our first prediction, on both final memory tests, participants’ improvement scores for studied versus unstudied items ranged from 25 to 40 percentage points for new facts (episodic memory) and 10 to 20 percentage points for name-face associations (semantic memory). The app thus showed great utility for helping all participants retain new fact-based information and maintain preexisting memories for name-face associations over time.

Regarding group differences, young adults and healthy older adults did not differ on the final tests of either new facts or name-face associations. This lack of age difference is consistent with some prior research [33,34] and likely occurred because recognition tests are less cognitively demanding than free-recall tests [35], and therefore subtle age-related memory decline is not always detected. Healthy older adults also did not outperform people with MCI due to AD on the celebrity name-face association test as predicted, possibly because the participants were at near-ceiling performance, thus masking potential differences in memory abilities. Healthy older adults did outperform people with MCI due to AD on the baseline test of new facts and on studied items on the 2 final tests of new fact-based memory, as expected.

We also posed several RQs without a priori predictions. First, we asked whether there would be differences in the magnitude of the spaced retrieval memory benefit for our 3 groups. We found that all participants experienced equally large improvements in recognition for studied versus unstudied items. These results are more positive than those found in prior research in which spaced retrieval conducted during a single laboratory session was more effective for healthy older adults than for older adults with AD [36,37]. Our results suggest that a multisession, longer-term memory intervention may better level the playing field between these 2 groups.

Regarding our RQ about memory decay, we did not observe decay across any groups during the approximately 1 week that elapsed after app cessation. Furthermore, given that the average delay between test 1 and test 2 was 8.30 (SD 1.99) days in practice, our observed results are even stronger than we anticipated, showing that memory for new facts and name-face associations was resilient to decay for slightly longer than 1 week. The lack of group differences is consistent with prior research showing that people with AD show similarly low rates of forgetting for well-learned information as healthy older adults [38]. In the real-life context of a person with early-stage AD, our results suggest that they could occasionally take a week off from practicing spaced retrieval without fear of substantial memory loss.

Finally, we asked whether the rate of learning would differ for healthy older adults and people with MCI due to AD. Our results showed no statistical difference in learning rates between the 2 groups for the semantic stimuli, but people with MCI due to AD needed more practice time than healthy older adults to master the episodic stimuli. These group differences were evident in the app use statistics: people with MCI due to AD spent more time using the app and answered more questions incorrectly; for example, across 4 weeks, healthy older adults averaged 1.4 minutes of use per day, whereas people with MCI due to AD averaged approximately 3.4 minutes of use per day. Altogether, our results suggest that people with MCI due to AD can achieve the same memory benefits from spaced retrieval as healthy older adults, but it takes them longer, and they must work harder to achieve these results.

Our results are also impactful when compared to the impact of pharmacological treatments for MCI due to AD. The most common class of drugs prescribed to manage cognitive symptoms in cases of MCI are ChEIs. Comprehensive meta-analyses suggest small [1,3] (standardized mean differences [SMDs] ranging from 0.22 to 0.55) or null [2] effects of ChEIs on a variety of common tests of cognitive function. Furthermore, the side effects of ChEIs, which include dizziness and digestive issues, can outweigh their modest benefits [2,4]. Recently, Food and Drug Administration–approved disease-modifying medications for AD that target amyloid beta using monoclonal antibodies [39] (SMDs ranging from –0.10 to 0.04) also showed small benefits for cognitive function in people with MCI due to AD, and their side effects can be even more serious [40]. By contrast, in our sample of people with MCI due to AD, using the app yielded SMDs of 2.32 (95% CI 1.46-3.16) for memory for new facts and 0.87 (95% CI 0.35-1.38) for memory for name-face associations on test 1, both large effects. Critically, these long-term memory improvements were achieved without any reported negative side effects. Apps that support memory retention cannot replace medication and certainly do not have the more global symptom–reducing effects of medication, but they may offer affordable, low-risk support for maintaining memory for specific, targeted information in AD.

Although this study mainly focused on the benefits of the mobile app for people with MCI due to AD, the results regarding healthy older adults should not be overlooked. Compared to young adults, healthy older adults showed statistically equivalent recognition memory for both new facts and name-face associations. Memory does decline in older age [35], and many older adults understandably worry about their memory. Using a spaced retrieval app could offer beneficial memory support.

### Limitations

Some aspects of the design of this study limit its generalizability to the real lives of individuals living with AD. First, the stimuli used in this study map well onto certain types of memory, such as learning new facts and recalling names of familiar people, but more research is needed to determine whether other types of content of long-term episodic and semantic memory are equally well supported. Examples of episodic memories that might be trained using the app include details of recent autobiographical events or facts associated with a recently learned skill (eg, learning to read music). Examples of semantic memories that could be trained include memory for sequences of numbers (eg, social security number and telephone numbers of loved ones) or ordered tasks that were once well known (eg, the correct steps for using one’s washing machine).

Second, it remains to be determined whether the benefits of spaced retrieval that we observed can transfer to free-recall tasks; for example, we found that using the app helps people with MCI due to AD recognize a familiar name on a list, but it is unknown whether they could recall this name without any prompt. Future research could include free-recall memory tests and may consider using free-recall–style questions as part of the spaced retrieval training itself. If the goal of people with AD is to freely recall information, a well-supported theory of memory (ie, *transfer-appropriate processing*) [41] would suggest that they should practice free recall when studying. In addition, future research is needed to determine whether using spaced retrieval to improve memory for specific information can confer more general long-term memory benefits, beyond simply remembering the studied information itself.

Third, the 5-week duration of this study limited the conclusions we could draw regarding memory durability and individuals’ adherence to the app over longer periods of time. On the former point, research suggests that the benefits of spaced retrieval for people with AD may not begin to fade until several weeks after cessation [42], a finding we could not replicate due to the limited time frame of our study. Future research should investigate the durability of memories in people with AD after longer periods of time away from the support of spaced retrieval. Conversely, studies in which people with AD continue to use a spaced retrieval app for a longer period would speak to their ability to adhere to it over many months or years.

Fourth and last, the generalizability of this study was limited by the size and demographic makeup of our sample. Although our sample of 20 people with MCI due to AD was both statistically powered and comparable to similar studies involving computer-based applications in people with AD [43-45], a larger sample would provide more demographic diversity. Our sample was disproportionately White and male. Considering that AD affects women more than men and Black and Hispanic individuals more than White individuals [46], future researchers should recruit a sample that more closely reflects the population of individuals living with AD. However, regarding gender differences, women typically outperform men on both verbal memory and face recognition tasks [47,48]. Therefore, we have no reason to expect that women would benefit less than men from this spaced retrieval intervention.

### Conclusions

Spaced retrieval has long been shown to support long-term memory retention in people with early-stage AD [5]. This pilot study demonstrated the efficacy and usability of a mobile app that serves as the vehicle for delivering spaced retrieval to these individuals, with no researcher support needed and little to no caregiver involvement required. These results come at a time when new disease-modifying therapies are likely to result in people with AD staying in the MCI and mild dementia stages longer. Given that pharmacological interventions that treat the symptoms of AD (eg, ChEIs) currently only offer small memory improvements to individuals with AD, augmentation with nonpharmacological treatments is critical. We have demonstrated one such assistive technology that can improve memory for fact-based information and name-face associations in people with MCI due to AD using an algorithm-driven, individualized spaced retrieval app requiring just minutes of use each day.

We will end on a practical note regarding how older adults with MCI due to AD and those without could expect to use this app in their daily lives. Our results suggest that all older adults could expect to spend 1 to 4 minutes a day answering questions on the app. After practicing a set of questions for 4 weeks, they could feel comfortable taking a week off from the app without experiencing noticeable memory decay. Furthermore, for the time investment of 1 to 4 minutes a day, older adults with MCI due to AD could expect to master approximately 45 pieces of information in 28 days. Healthy older adults could expect slightly faster mastery. Thus, all older adults could conservatively expect to commit approximately 45 pieces of information to memory in a month, and that information could consist of new facts, old memories for name-face associations that need refreshing, or both. If older adults were interested in spending >1 to 4 minutes using the app each day, they would likely be able to master even more information each month. Healthy older adults and many with MCI due to AD could easily navigate and adhere to the app on their own, whereas some older adults with MCI due to AD would need assistance from a caregiver.

## Appendix

Multimedia Appendix 1 Description of the Blank Slate app.

Multimedia Appendix 2 Means and, in parentheses, SEMs for episodic (facts about the country Georgia) and semantic (celebrity names) recognition memory on the final memory tests, test 1 and test 2.

## Abbreviations

AD: Alzheimer disease
BU ADRC: Boston University Alzheimer’s Disease Research Center
ChEI: cholinesterase inhibitor
MCI: mild cognitive impairment
MoCA: Montreal Cognitive Assessment
RQ: research question
SMD: standardized mean difference
VA: Department of Veterans Affairs

## Data Availability

The data sets generated and analyzed during this study are not publicly available because data generated by the Blank Slate app are considered proprietary but are available from the corresponding author on reasonable request.
