# Supplementary material for: Algorithmic Spaced Retrieval Enhances Long-Term Memory in Alzheimer Disease: Case-Control Pilot Study
Source: JMIR Form Res. 2024 Jul 19;8:e51943. doi: 10.2196/51943 (PMC11297374; doi:10.2196/51943)
Supplement: Multimedia Appendix 1 [file formative_v8i1e51943_app1.docx]

Appendix A

The Blank Slate app is a software platform that combines the learning technique called spaced retrieval with a machine learning model to provide users with individualized memory support. Spaced retrieval involves repeatedly recalling information (i.e., retrieving) that one desires to learn after increasingly lengthy intervals of time (i.e., spacing).

For the user, a typical Blank Slate session involves engaging with the app for a few minutes per day to answer a series of questions on to-be-remembered topics. Questions can be presented in several formats (e.g., multiple-choice, true/false, and text-entry). After each response from the user, the app provides feedback regarding accuracy and, when a response is inaccurate, the app provides the correct answer (see screenshot below). During each app session, users must answer each question correctly one time before the session is over. After a session is finished, a finish screen appears and the user cannot engage with any of the questions again for 24 hours, when the next question set is made available.


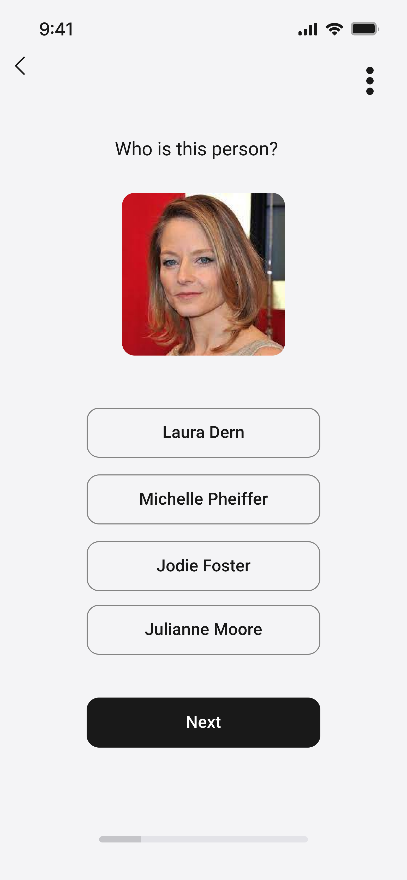

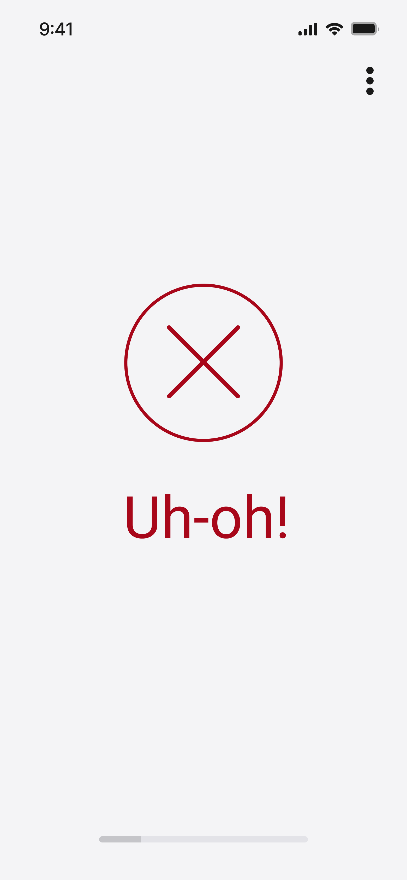

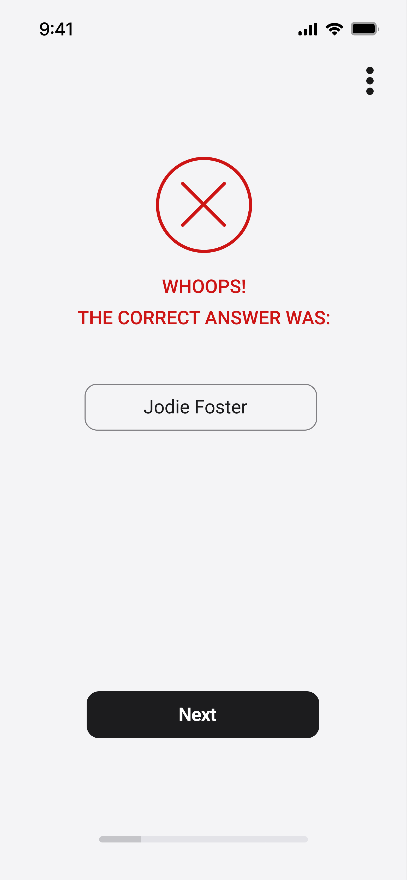


The app features a custom algorithm that predicts each user’s rate of forgetting for each question. During a given session, users review questions that they are most at risk of forgetting. If the app determines that users are not at a high risk of forgetting any questions on a given day, the app does not prompt users to engage with it at all. Thus, the app provides an individualized schedule of spacing, such that users review questions at spaced out intervals that are unique to their personal rate of forgetting. Blank Slate’s proprietary machine learning model was trained on over 500,000 observations and counting from the company’s earliest clients.

Blank Slate will comply with any requests to use their product for research purposes, including independent replication studies.
