# Supplementary material for: Algorithmic Spaced Retrieval Enhances Long-Term Memory in Alzheimer Disease: Case-Control Pilot Study
Source: JMIR Form Res. 2024 Jul 19;8:e51943. doi: 10.2196/51943 (PMC11297374; doi:10.2196/51943)
Supplement: Multimedia Appendix 2 [file formative_v8i1e51943_app2.docx]

Appendix B

Means and, in parentheses, SEMs for episodic (facts about the country Georgia) and semantic (celebrity names) recognition memory on the final memory tests, Test 1 and Test 2.

|  | **Episodic Memory** | | | | **Semantic Memory** | | | |
| --- | --- | --- | --- | --- | --- | --- | --- | --- |
|  | Test 1 | | Test 2 | | Test 1 | | Test 2 | |
|  | Unstudied | Studied | Unstudied | Studied | Unstudied | Studied | Unstudied | Studied |
| Young adults | 0.49 (.*03*) | 0.91 (.*02*) | 0.55 (.*03*) | 0.87 (.*02*) | 0.77 (.*03*) | 0.95 (.*02*) | 0.82 (.*04*) | 0.95 (.*01*) |
| Healthy older adults | 0.48 (.*03*) | 0.83 (.*03*) | 0.50 (.*03*) | 0.82 (.*03*) | 0.83 (.*04*) | 0.98 (.*01*) | 0.81 (.*05*) | 0.97 (.*01*) |
| People with MCI due to AD | 0.42 (.*02*) | 0.74 (.*04*) | 0.44 (.*03*) | 0.69 (.*04*) | 0.76 (.*05*) | 0.93 (.*02*) | 0.73 (.*05*) | 0.90 (.*03*) |
